# Health plan administrative records versus birth certificate records: quality of race and ethnicity information in children

**DOI:** 10.1186/1472-6963-10-316

**Published:** 2010-11-23

**Authors:** Ning Smith, Rajan L Iyer, Annette Langer-Gould, Darios T Getahun, Daniel Strickland, Steven J Jacobsen, Wansu Chen, Stephen F Derose, Corinna Koebnick

**Affiliations:** 1 Department of Research and Evaluation, Kaiser Permanente Southern California, Pasadena, CA, USA

## Abstract

**Background:**

To understand racial and ethnic disparities in health care utilization and their potential underlying causes, valid information on race and ethnicity is necessary. However, the validity of pediatric race and ethnicity information in administrative records from large integrated health care systems using electronic medical records is largely unknown.

**Methods:**

Information on race and ethnicity of 325,810 children born between 1998-2008 was extracted from health plan administrative records and compared to birth certificate records. Positive predictive values (PPV) were calculated for correct classification of race and ethnicity in administrative records compared to birth certificate records.

**Results:**

Misclassification of ethnicity and race in administrative records occurred in 23.1% and 33.6% children, respectively; the majority due to missing ethnicity (48.3%) and race (40.9%) information. Misclassification was most common in children of minority groups. PPV for White, Black, Asian/Pacific Islander, American Indian/Alaskan Native, multiple and other was 89.3%, 86.6%, 73.8%, 18.2%, 51.8% and 1.2%, respectively. PPV for Hispanic ethnicity was 95.6%. Racial and ethnic information improved with increasing number of medical visits. Subgroup analyses comparing racial classification between non-Hispanics and Hispanics showed White, Black and Asian race was more accurate among non-Hispanics than Hispanics.

**Conclusions:**

In children, race and ethnicity information from administrative records has significant limitations in accurately identifying small minority groups. These results suggest that the quality of racial information obtained from administrative records may benefit from additional supplementation by birth certificate data.

## Background

Increasing attention has been given to the research potential of information collected in electronic health records [1-3]. Electronic health records have been successfully used to improve patient care [4-6]. Electronic health records also help to obtain important information on demographic and behavioural characteristics, medical conditions, and health care costs [7-10]. Among the most burning questions is the understanding of racial and ethnic disparities in health care utilization and their underlying causes [11,12]. To address these problems, valid race and ethnicity information is needed.

Many health plans collect race and ethnicity information from their members [13,14]. This data comes from various sources such as insurance enrollment forms, inpatient and outpatient visit information, and birth certificates while the quality varies from different sources. Some studies indicated that the quality of this administrative data is fairly good in adults [14,15] but have some limitations for small minority groups such as American Indians [15]. The quality of race and ethnicity information for children, however, is largely unknown. Relatively frequent medical visits at a young age in children accompanied by a parent may result in higher quality of race and ethnicity information for children with adults.

Information from birth certificates is considered a criterion standard because it is nearly universal, includes self-reported race and ethnicity, and has been frequently validated [16-19]. While race and ethnicity information from birth certificates has been shown to provide a valid data source with positive predictive values (PPV) for most races above 96%, known limitations exist for Native Americans [16].

To fill the knowledge gap on the quality of race and ethnicity information for children in the administrative records of integrated health care systems, we compared information from these administrative records of a large managed health care system to the maternal and paternal race and ethnicity information obtained from birth certificates. We also investigated the main sources of racial and ethnic misclassification and the effect of health care utilization on the quality of race and ethnicity information, taking into consideration that information in the electronic health record of an integrated health care system is constantly updated.

## Methods

### Study design and population

Kaiser Permanente Southern California (KPSC) is an integrated health care system that provides health care for approximately 3.3 million members in southern California. The coverage area of KPSC includes 10 counties with approximately 22.7 million residents (based on 2008 estimates). Thus, KPSC members represent about 16% of the underlying population. Members receive medical care in KPSC owned hospitals and medical offices in the southern California area. On average, about 30,000 children are born in KPSC hospitals each year. For the present study, we identified 357,389 children who were delivered in KPSC hospitals between January 1, 1998 and December 31, 2008. We excluded 31,579 (8.8%) children because the maternal race was missing on the birth certificate, resulting in a final study population of 325,810 children. The study protocol was reviewed and approved by the Institutional Review Board of KPSC.

### Race and ethnicity information from birth certificate records

Race and ethnicity from birth certificates are often used for federal statistics, particularly for intercensal population estimates and annual statistical tabulations regarding maternal and child health [20-22]. As all children are born in KPSC owned hospitals, birth certificate information is collected by clerks during the hospital stay and based on parental self-report. Information on maternal and paternal race from the KPSC birth certificate record database was used as the criterion standard to classify children as White, Black, Asian/Pacific Islander (PI), American Indian/Alaskan Native (AIAN), other race, or multiple races based on maternal and paternal race information. If the maternal and paternal race were not identical, children were classified as multiple races. The paternal race from birth certificates was unknown in 20.5% of the children. These children were classified according to the maternal race information obtained.

An infant's ethnicity was classified as Hispanic or non-Hispanic based on maternal and paternal ethnicity information obtained from birth certificates. If at least one parent was of Hispanic ethnicity, the infant was classified as Hispanic. Paternal ethnicity was unknown in 7.3%. These children were classified according to maternal ethnicity information. Maternal ethnicity was unknown in 53 children (<0.01%) who were classified based upon paternal information, or classified as unknown.

### Race and ethnicity information from health plan administrative records

Racial categories from health plan administrative records are collapsed to White, Black, AIAN, Asian/PI, multiple races, other races, and unknown/missing races. Ethnic categories are Hispanic and non-Hispanic. Information on race, ethnicity, and language preference is collected at health plan enrollment, as well as during inpatient and outpatient medical visits. These are referred to as administrative records. Medical staff is asked to update these administrative records and, therefore, information can change over time. For the present study, information on race and ethnicity was extracted as of Dec 31, 2008. Administrative records include information from three different sources using the most recent information: (1) The Kaiser Foundation System, which is a management information system for health plan administration and accounting; (2) the electronic health record (EMR) system HealthConnect; and (3) a hospital inpatient information system which was used before EMRs were implemented. No information from birth certificates was included in this source. Within these sources, language preference for medical visits and other contacts provided by the patient or guardian was used to supplement this information. A KPSC member is classified as Asian/PI race if any Asian language is preferred. A KPSC member is classified as Hispanic if any Spanish language is preferred. If the three informational sources deliver contradictory information on race (other than unknown information), the race is classified as multiple.

### Statistical analysis

We calculated racial/ethnic distribution of the study population based on birth certificates as the criterion standard compared with administrative records. We also calculated sensitivity (the conditional probability that a specific race/ethnicity according to birth certificates is correctly classified as such in administrative records) and positive predictive value (PPV) which is the proportion of children who are correctly classified in administrative records as being of a specific race/ethnicity among all children with this race/ethnicity). Sensitivity was calculated with and without subjects who have missing race/ethnicity information in the administrative records to distinguish between misclassification of race/ethnicity and misclassification due to non-classification. The distribution of unknown/missing race in the administrative records was comparable among most races/ethnicities (12.5-15.5%) except for AIAN (22.8%). Multivariable logistic regression models were used to estimate the relationship of correct classification of race/ethnicity with the length of health insurance coverage, number of medical encounters, and race and ethnicity. Odds ratio (OR) and their corresponding 95% confidence intervals (CI) are given. Statistical software package PASW Statistics 17.0 was used (SPSS Inc., Chicago, IL).

## Results

The majority of children (n = 174,361) enrolled in the study were Hispanic (Table 1). Regardless of ethnicity, 69.5% of children were classified as White (n = 240,214), 10.6% Black (n = 37,056), 9.7% Asian/PI (n = 40,895), 0.2% AIAN (n = 1,390), 0.3% of other race (n = 1,514), and 9.7% of multiple race (n = 4,741) based on maternal and paternal race from birth certificates.

**Table 1:** Characteristics of the study population

	Children
	(n = 325,810)
Age in 2008 (y)^1^	5.7 (2.5-8.4)
Membership duration (y)^1^	2.9 (1.0-6.1)
Medical encounters (n)^1^	20 (9-35)
Emergency	0 (0-2)
Inpatient	1 (1-1)
Stillbirth, neonatal death, or other non-live births (%)	1.3
Hispanic ethnicity (%)	53.5
Race (%)	
White	69.5
Black	10.6
Asian/PI^2^	9.7
AIAN^2^	8.1
Other race	0.3
Multiple races	9.7

All data are given as median (25^th ^- 75^th ^percentile) or percent.

^1 ^Age, membership duration, and number of medical encounters at Dec 31^st^, 2008

^2 ^Asian/PI: Asian or Pacific Islander, AIAN: American Indian or Alaskan Native

### Identification of Hispanic Ethnicity in Administrative Records

According to administrative records, 43.1% of children were Hispanic, 48.2% non-Hispanic, and 8.8% had an unknown ethnicity. Sensitivity and PPV for Hispanic ethnicities were 76.9% and 95.6%, respectively (Table 2). Because most cases of misclassification were due to missing ethnicity information in administrative records (48.3% of misclassified cases), we also calculated sensitivity without those with missing information. If children with unknown ethnicity in administrative records were excluded, the sensitivity was 84.7%.

**Table 2:** Sensitivity and positive values for racial/ethnic information from administrative and birth certificates records in children

	Sensitivity (95%-CI)		Positive predictive value
		
	Records with unknown race/ethnicity		
	included	excluded	(95%-CI)
**All Children (n = 325,810)**			
***Ethnicity:***			
Hispanic	76.9 (76.7-77.1)	84.7 (84.5-84.8)	95.6 (95.5-95.7)
***Race:***			
White	73.0 (72.8-73.2)	84.7 (84.6-84.9)	89.3 (89.2-89.5)
Black	80.6 (80.2-81.0)	92.6 (92.3-92.8)	86.6 (86.2-87.0)
Asian/Pacific Islander^1^	69.9 (69.3-70.4)	81.8 (81.4-82.3)	73.8 (73.3-74.3)
AIAN^1^	15.0 (12.4-18.0)	19.4 (16.1-23.2)	18.2 (15.0-21.8)
Other	42.3 (39.2-45.5)	50.1 (46.6-53.3)	1.2 (1.1-1.4)
Multiple	2.8 (2.6-3.0)^2^	3.1 (2.9-3.4)^2^	51.8 (49.4-54.2)
**Non-Hispanic children (n = 151,396)**			
***Race:***			
White	84.7 (84.4-85.0)	96.3 (96.1-96.4)	84.3 (84.0-84.6)
Black	81.4 (80.9-81.8)	93.3 (93.0-93.6)	91.7 (91.4-92.0)
Asian/Pacific Islander^1^	71.5 (71.0-72.0)	83.0 (82.5-83.3)	78.8 (78.3-79.2)
AIAN^1^	13.9 (10.3-18.6)	20.1 (14.9-26.5)	13.0 (9.5-17.4)
Other	82.1 (62.4-93.2)	92.0 (72.5-98.6)	0.07 (0.8-1.1)
Multiple	3.5 (3.2-2.8)	4.0 (3.7-4.3)	48.3 (45.5-51.1)
**Hispanic children (n = 174,361)**			
***Race:***			
White	67.6 (67.3-67.8)	79.2 (79.0-79.5)	92.5 (92.3-92.6)
Black	67.1 (65.0-69.2)	78.8 (76.7-80.8)	40.1 (38.4-41.8)
Asian/Pacific Islander^1^	44.9 (42.6-47.2)	60.8 (58.2-63.4)	28.8 (27.2-30.5)
AIAN^1^	15.8 (12.2-20.1)	18.9 (14.7-23.9)	25.5 (19.9-31.8)
Other	41.2 (38.8-44.4)	48.8 (45.3-52.3)	1.3 (1.2-1.5)
Multiple	1.9 (1.7-2.1)	2.1 (1.9-2.4)	62.0 (57.3-66.6)

^1 ^Asian/PI: Asian or Pacific Islander, AIAN: American Indian or Alaskan Native

^2 ^Counting infants as correctly classified when at least one parent's racial information was correctly identified, sensitivity for infants of multiple race increases to 74.4% when including and 90.2% when excluding unknown race in administrative records.

Identification of Hispanic ethnicity in administrative records was better in children whose parents were both Hispanic as compared to children with only one Hispanic parent (p < 0.001). In administrative records, 87.6% of children with parents who were both non-Hispanic or both Hispanic were identified in accordance with birth certificates. From children with only one Hispanic parent, children with Hispanic mothers (66.2%) were more likely to be identified as Hispanic in administrative records then children with Hispanic fathers (26.9%, p < 0.001).

Correct identification of Hispanic ethnicity was positively associated with the duration of health insurance coverage (OR per year of health insurance coverage 1.03, 95%-CI 1.02-1.04) but not with the number of all medical encounters. However, among medical encounters, inpatient (OR for each additional encounter 1.22, 95%-CI 1.19-1.25) and emergency (OR for each additional encounter 1.17, 95%-CI 1.16-1.18) visits showed a strong association with correct classification of Hispanic ethnicity.

### Identification of Race in Administrative Records

According to administrative records, 56.8% of children were White, 9.8% Black, 9.2% Asian/PI, 0.2% AIAN, 9.8% of other race, 0.5% of multiple race, and 13.7% of unknown race. The overall sensitivity was 66.4%, but this was higher in the three largest racial groups (Table 2). The low sensitivity was mainly caused by high numbers of children with unknown or missing race/ethnicity in administrative records (40.9% of misclassified cases). When children with unknown race in administrative records were excluded, the overall sensitivity increased to 86.7%.

The sensitivity and PPVs were lowest in children of multiple races. Among incorrectly classified children of multiple races, 56.2% of children were not identified correctly because only the maternal race and 17.5% because only the paternal race was recorded. If children with known race are counted as correctly classified when at least one parent’s racial information was reflected correctly, the overall PPV increased to 95.7% with a sensitivity of 90.2%.

**Figure 1:**
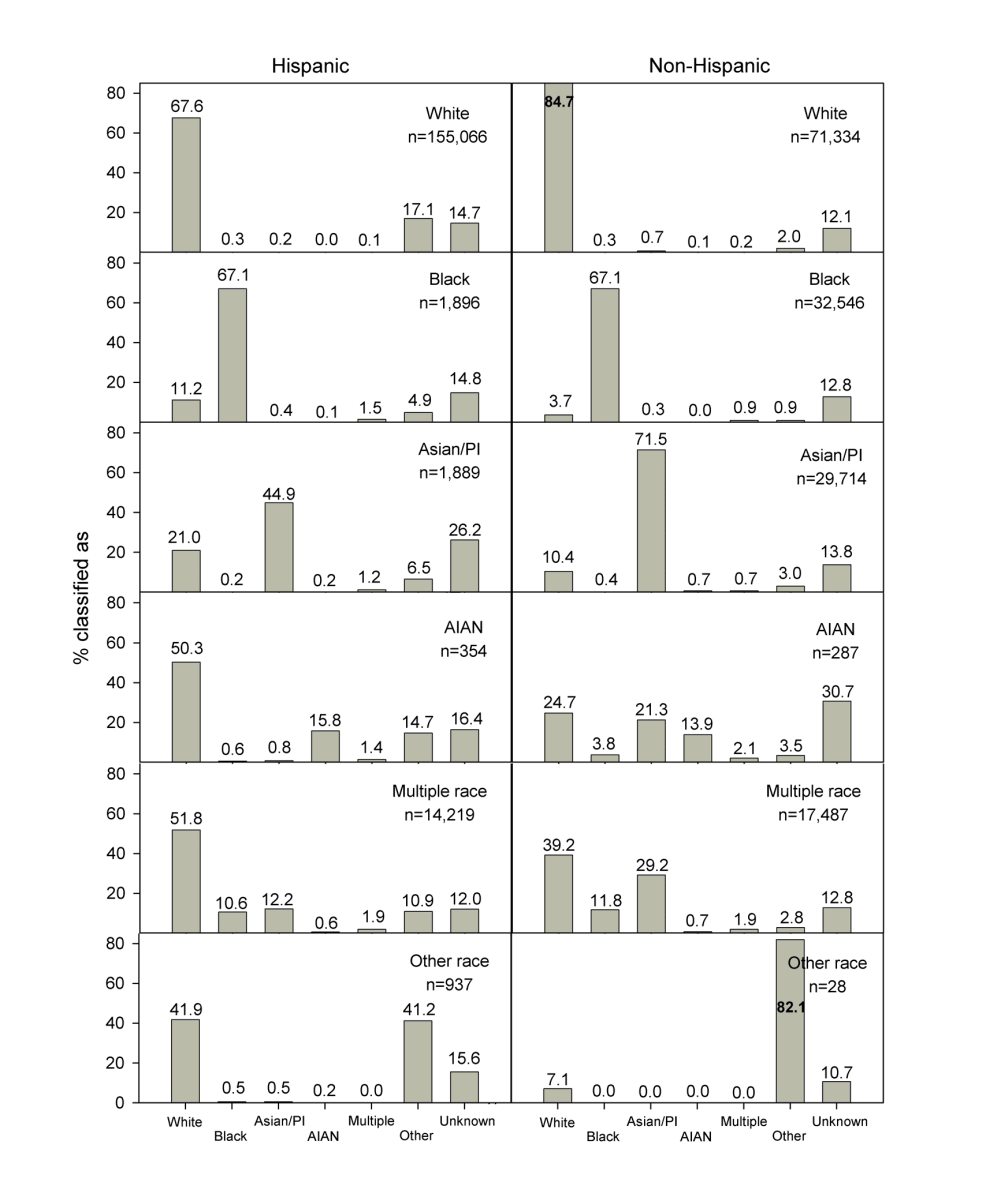
**Patterns of racial classification in administrative records from Hispanic (n = 174,361) and non-Hispanic (n = 151,396) children**. Abbreviations used: Asian/PI: Asian or Pacific Islander, AIAN: American Indian or Alaskan Native.

Correct identification of race varied by ethnicity (Table 2), the number of medical encounters, and birth outcome. The odds ratio for correct identification of race in the administrative records was higher in non-Hispanics (OR 2.62, 95%-CI 2.56-2.68) than in Hispanics. Stillbirth (OR 0.005, 95%-CI 0.004-0.006) but not neonatal death (OR 0.99, 95%-CI 0.72-1.38) decreased the odds for correct race identification. The total number of medical encounters only slightly increased the odds for correct race identification (OR for each additional encounter 1.01, 95%-CI 1.00-1.01). Among all medical encounters, inpatient (OR for each additional encounter 1.32, 95%-CI 1.30-1.35) and emergency (OR for each additional encounter 1.09, 95%-CI 1.09-1.10) visits showed the strongest association with correct race classification. Duration of health insurance coverage was not associated with the odds for correct race identification. Patterns of racial classification deviating from birth certificates (i.e. misclassification) differed significantly among non-Hispanics and Hispanics (Figure 1). When children with unknown race in administrative records were excluded, racial classification was more accurate in non-Hispanic Whites, Blacks, and Asians (PPV 81%). Hispanic children from minority groups were frequently misclassified as White.

## Discussion

This study utilized the most recent race and ethnicity data collected as part of the administrative records of a large, integrated health plan and compared it to information available from birth certificates. The information was accurate for ethnicity and for the three largest racial groups (White, Black, and Asian). Two major causes of disagreement between administrative and birth certificate records were identified: (1) missing information in administrative records and (2) classification of children of multiple races based on information from only one parent. Eliminating these causes would increase the sensitivity for correct racial classification from 66.4% to 95.7%. Because race and ethnicity information in health plan administrative records are constantly updated, information was more accurate in children with more medical encounters. Sensitivity and PPVs were generally higher in non-Hispanics than in Hispanics. Limitations in data quality were noted for children of multiple races and children of AIAN origin.

The quality of racial and ethnic information in children has not been well studied. However, the results from the present study were comparable to two previous studies investigating race and ethnicity information in adults [14,15]. In these studies, PPVs for Whites and Blacks were between 86.7% and 95.1%. However, PPVs and sensitivity for small minority groups such as AIAN were generally poor [15]. Comparably, PPV and sensitivity for Hispanic adults was lower than for non-Hispanic Whites and Blacks. These patterns are generally consistent with the accuracy observed for racial and ethnic information in Medicare enrollment databases [23]. The present study also shows that the patterns of misclassification varied greatly between Hispanic and non-Hispanic children.

In the present study, one major reason for race/ethnicity misclassification in the administrative records was missing information (non-classification). After exclusion of non-classified individuals, the sensitivity improved significantly for Whites, Black, and AIAN. This partially explains the lower sensitivity observed in our study compared to other studies which excluded non-classified individuals from their study population [14,15]. Incomplete and missing information on race, ethnicity and language in databases from health care organizations has been reported by others previously [24]. The results from our study suggest that birth certificate information is not routinely used to fill missing information in administrative records, even if available as in this setting.

The second important cause of disagreement between administrative records and birth certificates was the misclassification of children whose parents had a different race (i.e. multiple races). Among children of multiple races, the vast majority of children were misclassified because only racial information of one parent - mostly maternal information - was used for classification purposes. One possible explanation for this misclassification is an often observed simplification of multiracial heritage. Multiple races are often reported as one main race [25,26]. Multiracial identification varies across regions and races; in particular, AIAN are less likely to report themselves as multiracial [26]. It may also be speculated that maternal presence during birth as well as later medical encounters account for this observation.

The present study adds new information on changes in the quality of race information over the course of membership. Race and ethnicity data collected in an integrated health care system used in the present study are updated during medical visits, as opposed to other settings such as health insurance claims where race/ethnicity information is usually collected at enrollment. The present study shows that the quality of information increased over time with increasing number of medical encounters, especially inpatient visits. Although the effects may differ in magnitude by organization, we can assume our results are generalizable to other integrated health care settings that update their patient's demographic data during office visits.

Our study benefited from the substantial size of a diverse population with adequate numbers of Hispanic and non-Hispanic racial and ethnic group representation to generate ample statistical power and allow valid estimates of sensitivity and PPVs. A limitation of the present study is the use of information obtained from birth certificate records as a criterion standard. After carefully reviewing the birth certificate records, previous studies have reported that birth certificate records provide relatively valid information on race and ethnicity [16,19]. Race and ethnicity from birth certificates are also used as standards for federal statistics such as intercensal population estimates [20-22]. Despite PPVs of 96% and above for most races, significant limitations of the data quality were described for individuals of AIAN origin.

Consequences of misclassification of racial and ethnic minorities can lead to data misinterpretation and erroneous conclusions. Incorrect classification of individuals of a small minority group may lead to over or underestimation of health disparities and race-related risk factors. Therefore, accurate racial and ethnic information is crucial for health care research.

## Conclusions

Results of the present study suggest that the overall quality of racial and ethnic information is relatively good for distinguishing between Hispanics and non-Hispanics, Whites, and Blacks. Our results also show that use of health plan administrative records alone leads to frequent misclassification of minority groups and individuals of multiple races. Thus, linking birth certificate information to the administrative records of children can optimize the accuracy of race and ethnicity classification if this information is available.

## Competing interests

The authors report no conflicts of interest. The authors alone are responsible for the content and writing of the paper.

## Authors' contributions

Conception and design of the study and: NS, RY, CK

Acquisition of the data: NS, RY, CK

Analysis and interpretation: NS, RY, ALG, DG, DS, SJJ, WC, SD, CK

Drafting and critically revising the manuscript: NS, RY, ALG, DG, DS, SJJ, WC, SD, CK

Final approval: NS, RY, ALG, DG, DS, SJJ, WC, SD, CK

## Pre-publication history

The pre-publication history for this paper can be accessed here:

http://www.biomedcentral.com/1472-6963/10/316/prepub
